# Supplementary material for: Template-jumping prime editing enables large insertion and exon rewriting in vivo
Source: Nat Commun. 2023 Jun 8;14:3369. doi: 10.1038/s41467-023-39137-6 (PMC10250319; doi:10.1038/s41467-023-39137-6)
Supplement: Supplementary file 1 — Supplementary information [file 41467_2023_39137_MOESM1_ESM.pdf]

Template-jumping prime editing enables large insertion and exon rewriting *in vivo*

Chunwei Zheng[1#], Bin Liu[1#], Xiaolong Dong[1], Nicholas Gaston[1], Erik J. Sontheimer[1-3*], Wen Xue[1-4*]

[1]RNA Therapeutics Institute, University of Massachusetts Chan Medical School, Worcester, MA 01605, USA.

[2]Department of Molecular Medicine, University of Massachusetts Chan Medical School, Worcester, MA 01605, USA.

[3]Li Weibo Institute for Rare Diseases Research, University of Massachusetts Chan Medical School, Worcester, MA 01605, USA.

[4]Department of Molecular, Cell and Cancer Biology, University of Massachusetts Chan Medical School, Worcester, MA 01605, USA.

# Equal contributions to this study

*Corresponding author: Erik J. Sontheimer (erik.sontheimer@umassmed.edu), Wen Xue (wen.xue@umassmed.edu)

Fig.S1

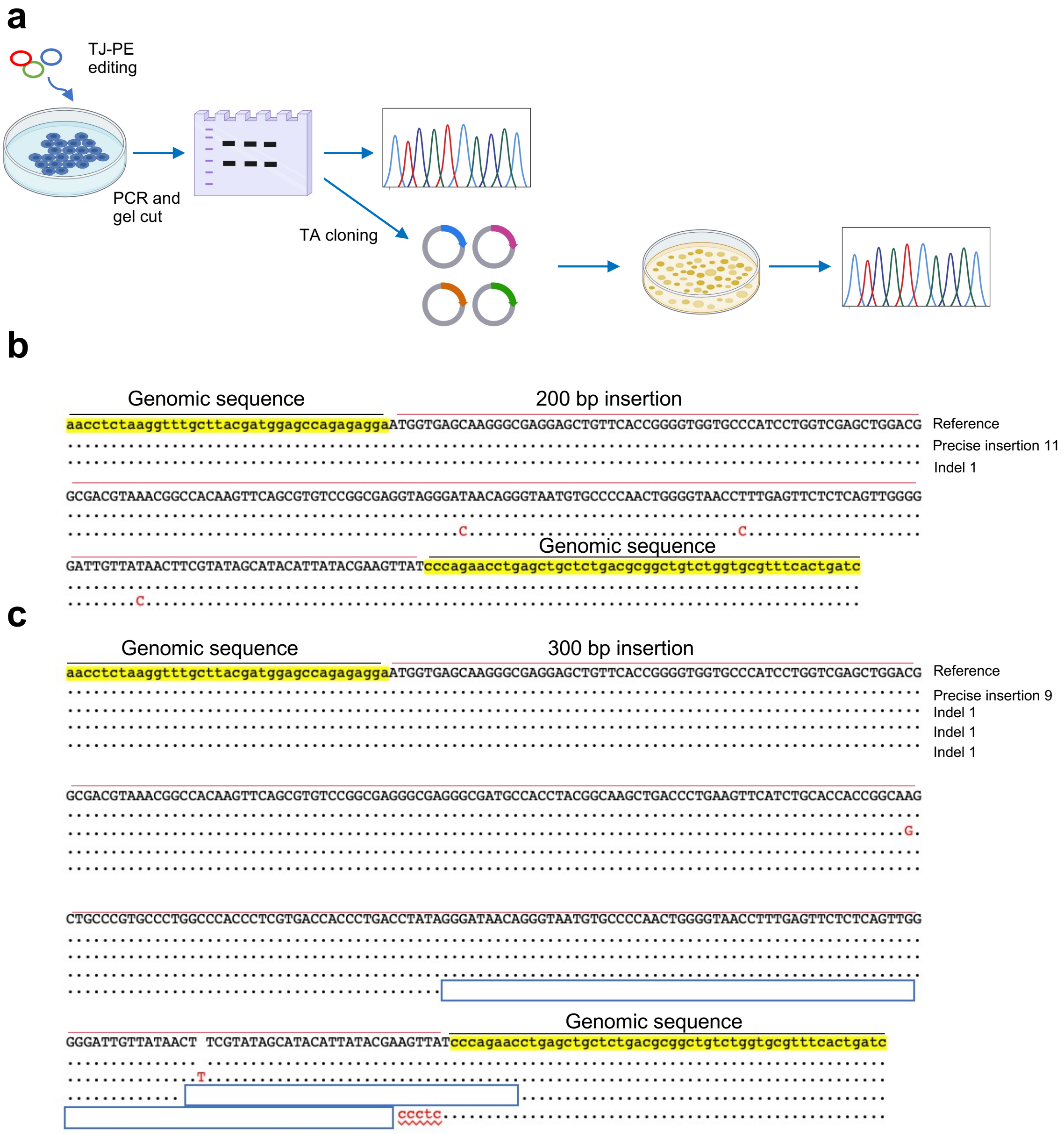
a
TJ-PE editing
PCR and gel cut
TA cloning
b
Genomic sequence
200 bp insertion
aacctctaaggtttgcttacgatggagccagagaggaATGGTGAGCAAGGGCGAGGAGCTGTTCACCGGGGTGGTGCCCATCCTGGTCGAGCTGGACG
Reference
Precise insertion 11
Indel 1
GCGACGTAAACGGCCACAAGTTCAGCGTGTCCGGCGAGGTAGGGATAACAGGGTAATGTGCCCCAACTGGGGTAACCTTTGAGTTCTCTCAGTTGGGG
C
C
Genomic sequence
GATTGTTATAACTTCGTATAGCATACATTATACGAAGTTATcccagaacctgagctgctctgacgcggctgtctggtgcgtttcactgatc
C
c
Genomic sequence
300 bp insertion
aacctctaaggtttgcttacgatggagccagagaggaATGGTGAGCAAGGGCGAGGAGCTGTTCACCGGGGTGGTGCCCATCCTGGTCGAGCTGGACG
Reference
Precise insertion 9
Indel 1
Indel 1
Indel 1
GCGACGTAAACGGCCACAAGTTCAGCGTGTCCGGCGAGGGCGAGGGCGATGCCACCTACGGCAAGCTGACCCTGAAGTTCATCTGCACCACCGGCAAG
G
CTGCCCGTGCCCTGGCCCACCCTCGTGACCACCCTGACCTATAGGGATAACAGGGTAATGTGCCCCAACTGGGGTAACCTTTGAGTTCTCTCAGTTGG
Genomic sequence
GGGATTGTTATAACT TCGTATAGCATACATTATACGAAGTTATcccagaacctgagctgctctgacgcggctgtctggtgcgtttcactgatc
T
ccctc

d

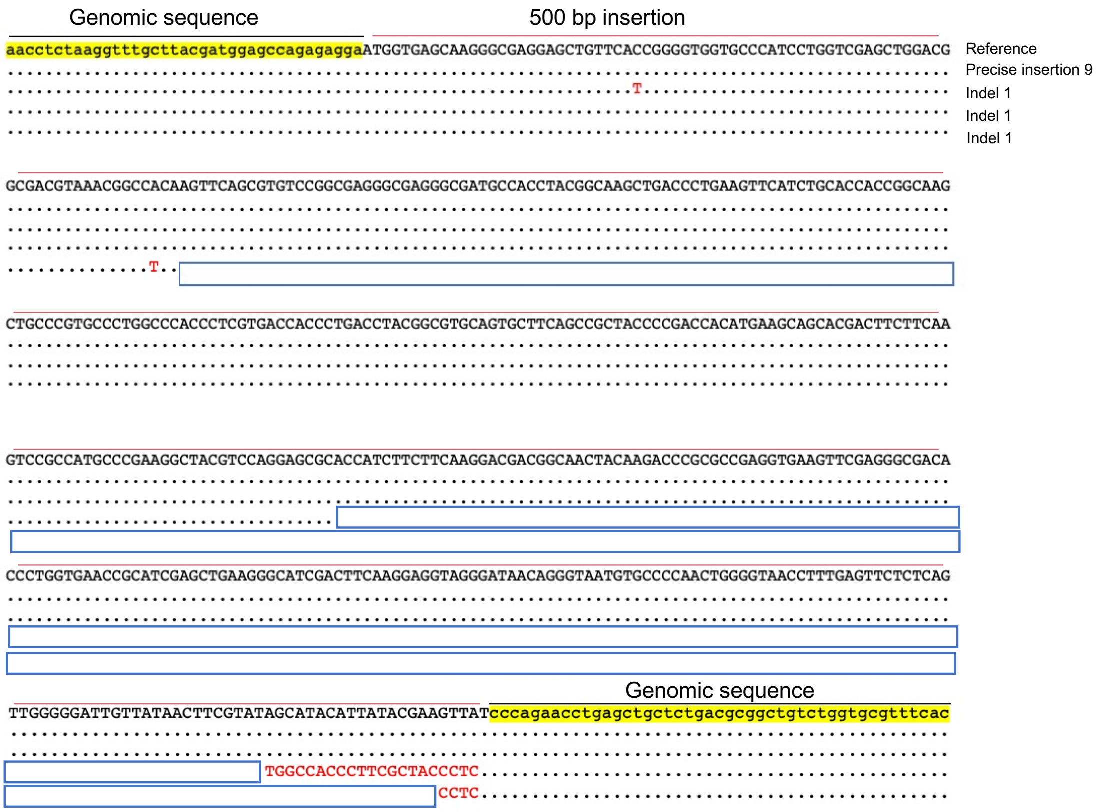

Supplementary Fig. 1. TJ-pegRNA achieved accurate large insertion at *AAVS1* locus. a, Schematic showing procedure for determining accuracy of 200-, 300-, and 500-bp insertions at the *AAVS1* locus. Insertion bands were cut out of the agarose gel and purified for Sanger sequencing and TA cloning. **b-d**, Sanger sequencing of single clone of **b**, 200 bp insertion **c**, 300 bp insertion **d**, 500 bp insertion at *AAVS1* locus. Deleted bases were indicated by blue box and mismatches relative to reference are in red.

Fig.S2

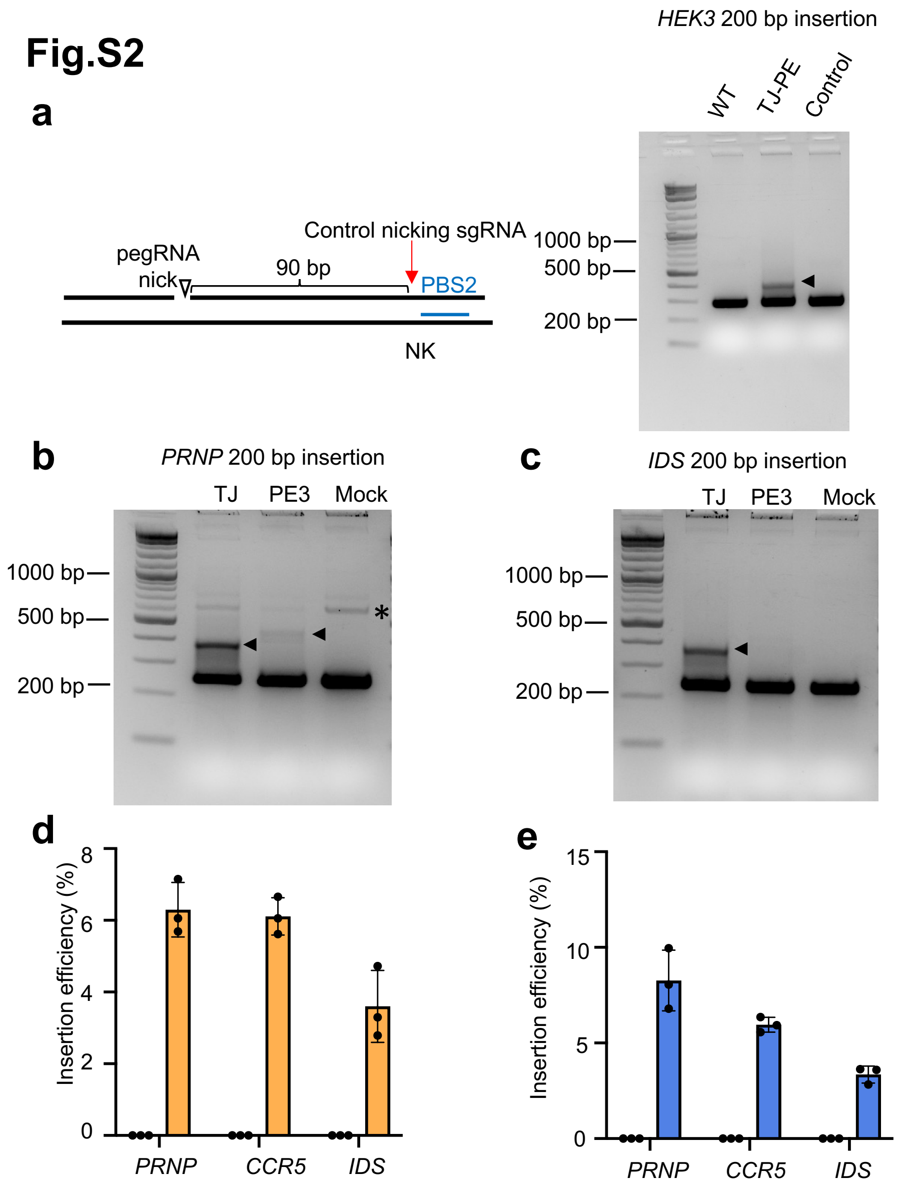

Supplementary Fig. 2. TJ-PE mediates insertions at multiple genomic loci and cell lines. a, New control nicking sgRNA designed to nick at the same position as nicking sgRNA, but on the opposite strand at the *HEK3* locus. The insertion band of the expected size was observed following TJ-PE treatment (arrow), but not controls. Experiments were done three times, and one is shown. **b**, Insertion of a 200-bp DNA fragment with concomitant deletion of 72 bp at *PRNP* locus in HEK293T cells. The insertion bands of expected size are indicated with arrows. The nonspecific bands are indicated with asterisk. Experiments were done three times, and one is shown. **c**, Insertion of a 200-bp DNA fragment with concomitant deletion of 70 bp at *IDS* locus. The insertion bands of the expected size are indicated with arrows. Experiments were done three times, and one is shown. **d,** Insertion of a 200-bp DNA fragment measured by ddPCR at multiple loci in U-2 OS cells. PE plasmid served as negative control. **e**, Insertion of a 200-bp DNA fragment measured by ddPCR at multiple loci in A549 cells. PE plasmid served as control. Results were obtained from three independent experiments, shown as mean ± s.d.

Fig.S3

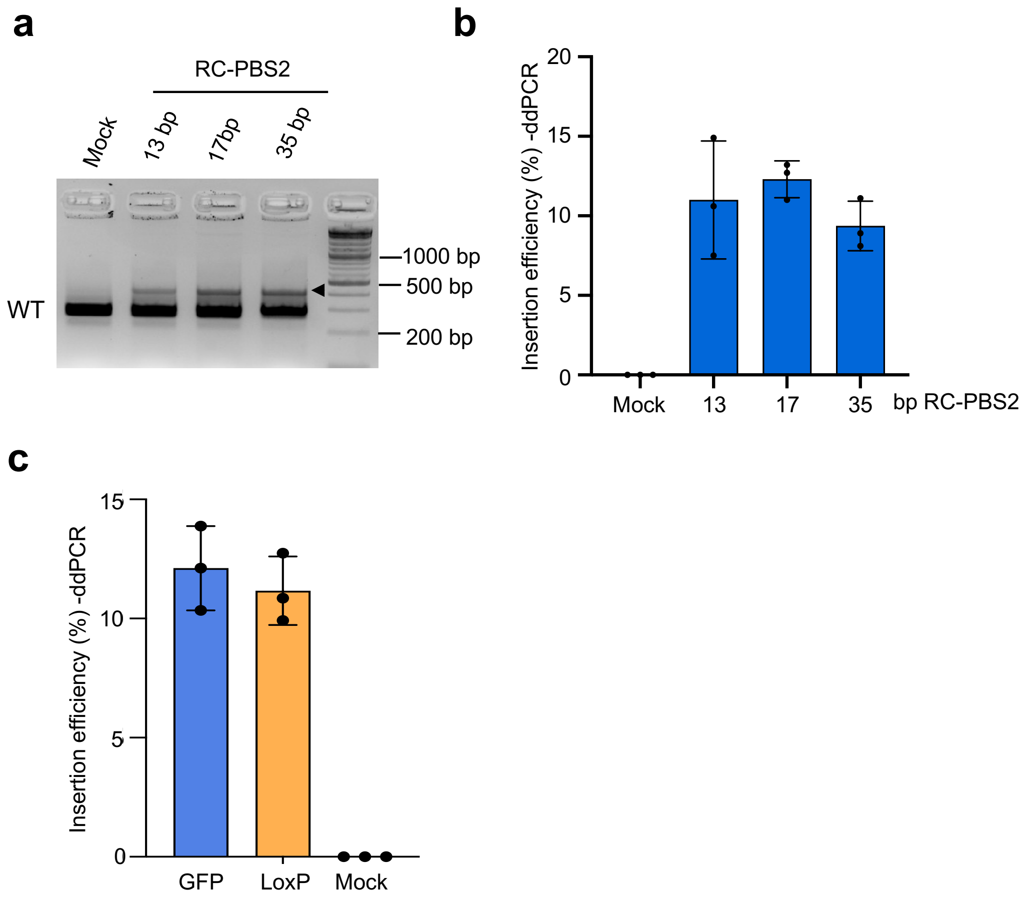

Supplementary Fig. 3. Comparison of the insertion efficiencies of the different lengths of PBS2 sequence or secondary structure. a, Agarose gel shows the insertion band of the expected size for TJ-pegRNA with 13, 17 or 35-bp RC-PBS2 at the *HEK3* locus (arrow). **b**, Insertion efficiency of a 200-bp DNA fragment with various lengths of PBS2 measured by ddPCR. **c**, Comparing insertion of GFP fragments without or with a hairpin (LoxP) at the HEK3 locus. Insertion efficiency was quantified by ddPCR. Results were obtained from three independent experiments, shown as mean ± s.d.

Fig.S4

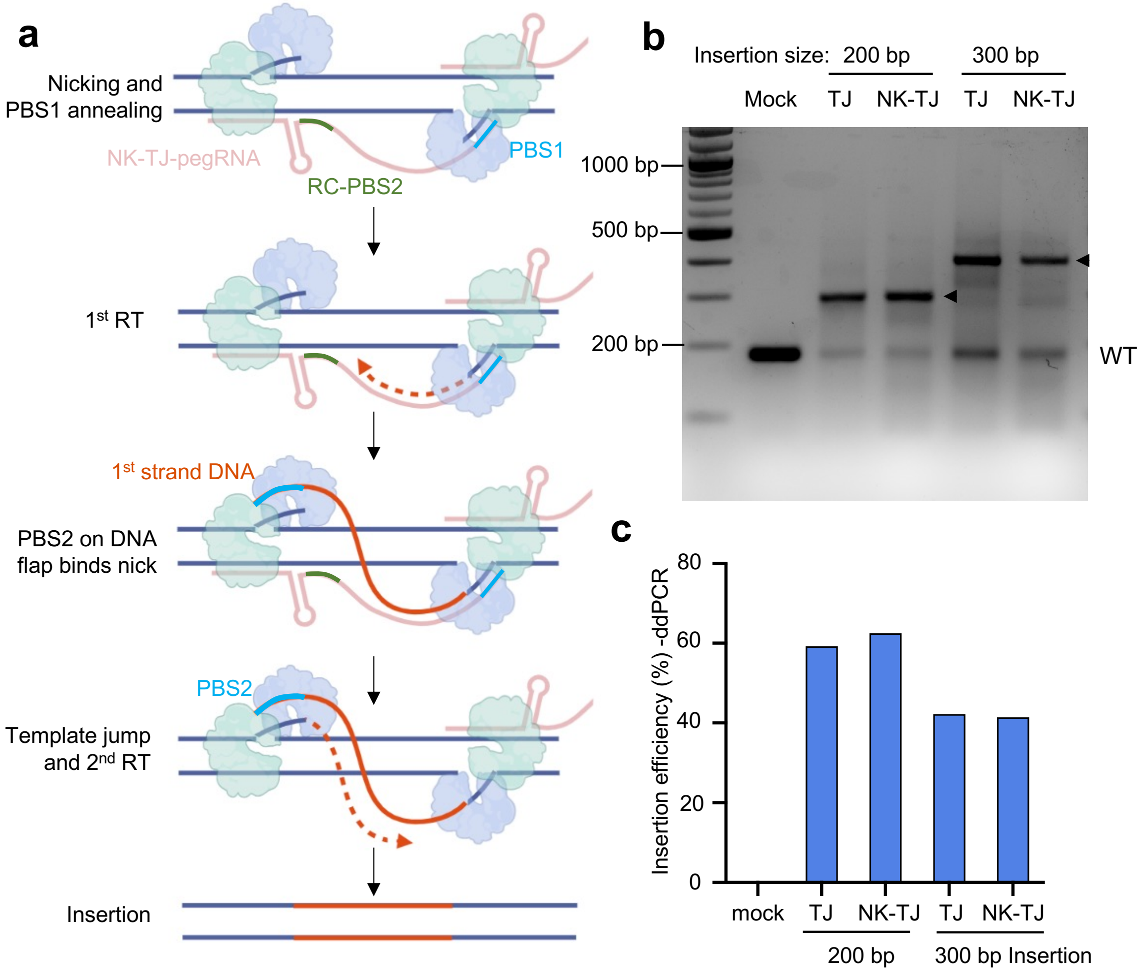

Supplementary Fig. 4. NK-TJ-pegRNA enables comparable insertion efficiency with TJ-pegRNA. a, Illustration of nicking-TJ-pegRNA (NK-TJ-pegRNA), which contains PBS1, RC-PBS2 and an insertion sequence. Compared to TJ-pegRNA, the PBS1 sequence of NK-TJ-pegRNA first hybridizes to the DNA flap generated by the nicking sgRNA. The newly synthesized PBS2 hybridizes to the second nicked site generated by NK-TJ-pegRNA to initiate the second strand synthesis. **b**, Agarose Gel shows insertion bands of expected sizes (200 bp and 300 bp) at *AAVS1* locus. **c**, Insertion efficiency quantified by ddPCR.

Fig.S5

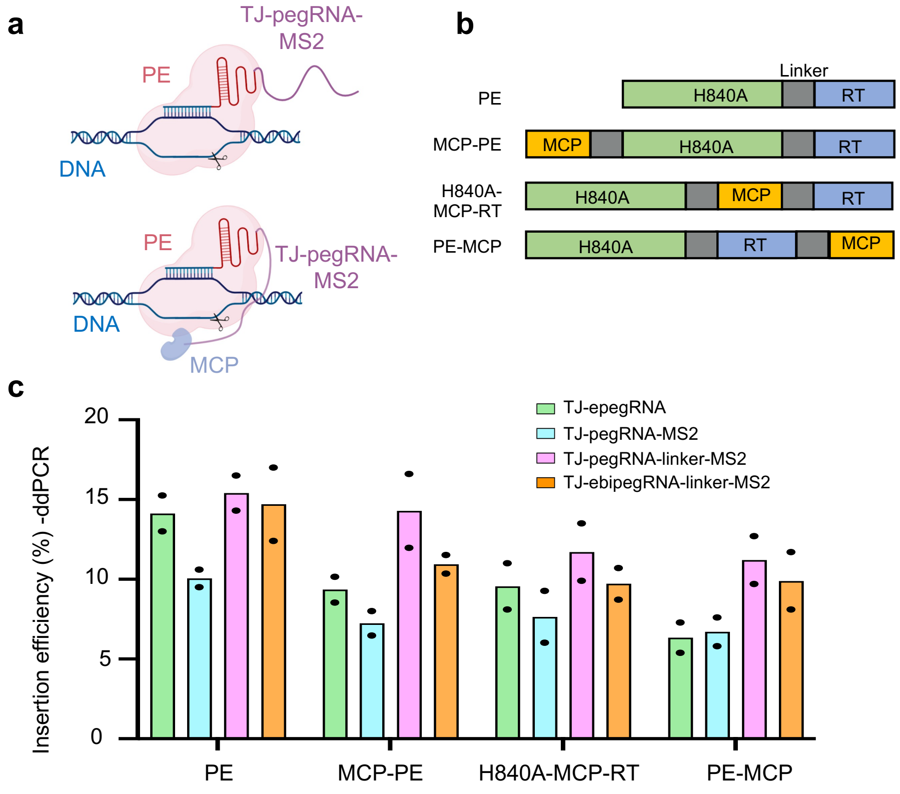

Supplementary Fig. 5. TJ-pegRNA with 3'-RNA aptamer. a, Diagram of pegRNA with 3'-RNA aptamer. **b**, Structure of the MCP and PE fusion protein. Schematic representations of PE-MCP fusion proteins. **c**, Insertion efficiency quantified by ddPCR at *HEK3* locus. Results were obtained from two independent experiments.

Fig. S6

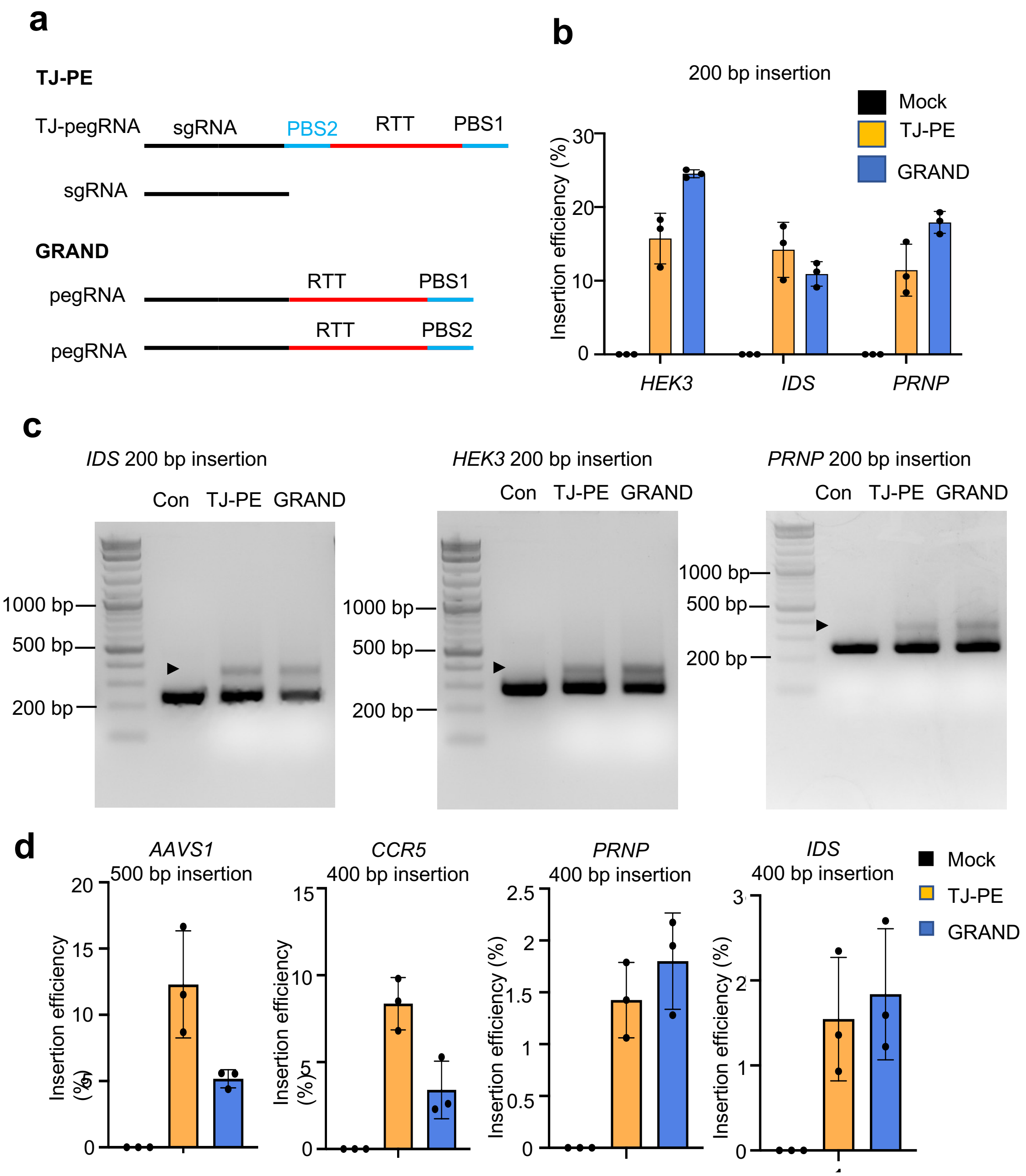

Supplementary Fig. 6. Comparison of insertion efficiencies mediated by GRAND and TJ-PE. a, Illustration of TJ-pegRNA and GRAND pegRNAs. **b**, Insertion of 200-bp DNA fragment with TJ-PE or GRAND editing at *HEK3*, *IDS* and *PRNP* loci in HEK293T cells. **c**, Agarose gel shows insertion bands of expected sizes (arrows). **d**, Insertion of DNA fragment at *AAVS1* (500-bp), *CCR5* (400-bp), *PRNP* (400-bp) and *IDS* (400-bp) loci. Insertion efficiency was quantified by ddPCR. Results were obtained from three independent experiments, shown as mean ± s.d.

Fig.S7

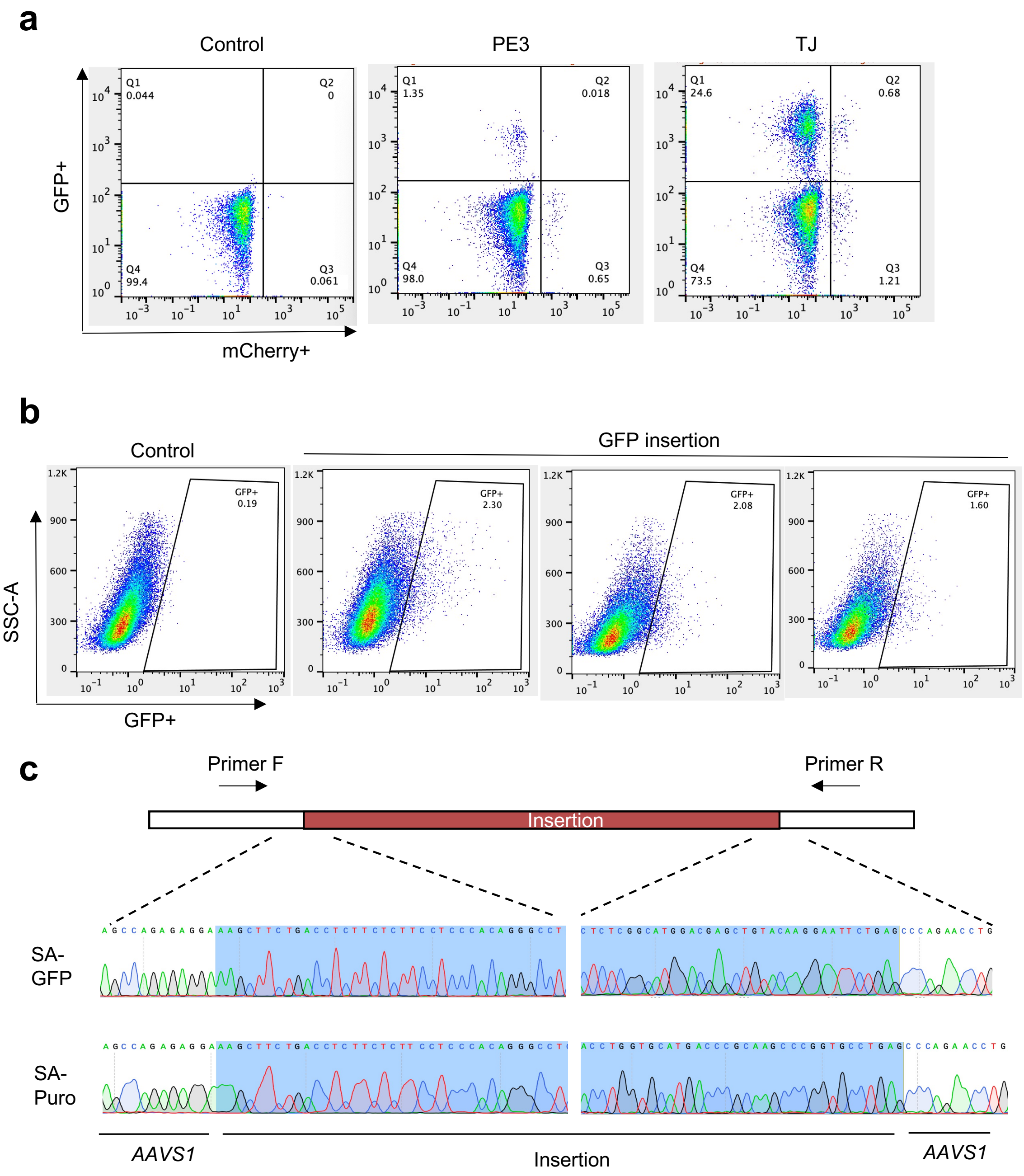

Supplementary Fig. 7. TJ-pegRNA mediates GFP reporter repair and functional gene insertion. a, Flow cytometry plots show the gating of TLR-MCV1 cells in control, PE3 and TJ-PE treated groups. **b**, Flow cytometry plots show the gating of GFP expression cells. **c**, Sanger sequence of SA-GFP and SA-Puro insertion. The insertion bands of predicted size were gel purified. Sanger sequencing shows precise insertions.

Fig.S8

a

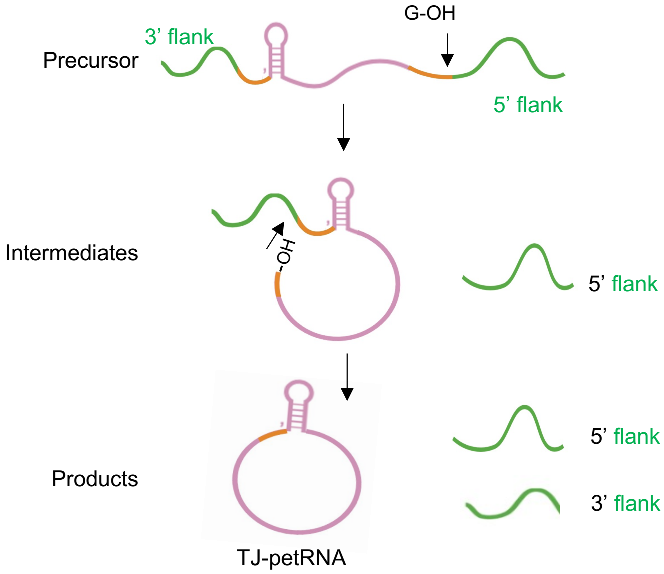

b

Full-length TJ-pegRNA

```
  ▼Cut site                                                                         Cut site▼
GGATAGGGATAACAGGGTAATGTGCCCCAACTGGGGTAACCTTTGAGTTCTCTCAGTTGGGGGATTGTTATAACTTCGTATAGCATACATTATACGAAGTTATCCCAGAACCTGAG   Insertion reference
GGATAGGGATAACAGGGTAATGTGCCCCAACTGGGGTAACCTTTGAGTTCTCTCAGTTGGGGGATTGTTATAACTTCGTATAGCATACATTATACGAAGTTATCCCAGAACCTGAG   0.4%    Precise

GGATCCTGGGAGGGAGAGCTTGGCAGGGGGTGGGAGGGAAGGGGGGGATGCGTGACCTGCCCGGTTCTCAGTGGCCACCCTGCGCTACCCTCTCCC                       WT reference
GGATCCTGGGAGGGAGAGCTTGGCAGGGGGTGGGAGGGAAGGGGGGGATGCGTGACCTGCCCGGTTCTCAGTGGCCACCCTGCGCTACCC------                       0.43%   Indels
```

Split circular TJ-petRNA

```
  ▼Cut site                                                                         Cut site ▼
GGATAGGGATAACAGGGTAATGTGCCCCAACTGGGGTAACCTTTGAGTTCTCTCAGTTGGGGGATTGTTATAACTTCGTATAGCATACATTATACGAAGTTATCCCAGAACCTGAG   Insertion reference
GGATAGGGATAACAGGGTAATGTGCCCCAACTGGGGTAACCTTTGAGTTCTCTCAGTTGGGGGATTGTTATAACTTCGTATAGCATACATTATACGAAGTTATCCCAGAACCTGAG   41.8%  Precise edits
GGATAGGGATAACAGGGTAATGTGCCCCAACTGGGGTAACCTTTGAGTTCTCTCAGTTGGGGGATTGTTATAAC-------------------------------------CTGAG   0.9%
GGATAGGGATAACAGGGTAATGTGCCCCAACTGGGGTAACCTTTGAGTTCTCTCAG----------------------------------------------------AACCTGAG   0.7%
GGATAGGGATAACAGGGTAATGTGCCCCAACTGGGGTAACCTTTGAGTTCTCTCAGTTGGGGGATTGTTATAACTTCGTATAGCATACATTATACGAA------------CCTGAG   0.7%
GGATAGGGATAACAGGGTAATGTGCCCCAACTGGGGTAACCTTTGAGTTCTCTCAGTTGGGGGATTGTTATAACTTCGTATAGCATAC-------------------GAACCTGAG   0.5%
GGATAGGGATAACAGGGTAATGTGCCCCAACTGGGGTAACCTTTGAGTTCTCTCAGTTGGGGGATTGTTATAACTTCGTATAGCATACA------------------GAACCTGAG   0.4%
GGATAGGGATAACAGGGTAATGTGCCCCAACTGGGGTAACCTTTGAGTTCTC----------------------------------------------------CCAGAACCTGAG   0.4%   Imperfect
GGATAGGGATAACAGGGTAATGTGCCCCAACTGGGGTAACCTTTGAGTTCTCTCAGTTGGGGGATTGTTATAACTTCGTATAGCATAC-----------------------CTGAG   0.3%   edits
GGATAGGGATAACAGGGTAATGTGCCCCAACTGGGGTAACCTTTGAGTTCTCTCAGTTGGGGGATTGTTATAACTTCGTATAGCATACATTATACGAAGTTATCCCAGAACCTGA    0.3%
GGATAGGGATAACAGGGTAATGTGCCCCAACTGGGGTAACCTTTGAGTTCTCTCAGTTGGGGGATTGTTATAACTTCGTATAGCATA-----------------CCAGAACCTGAG   0.3%
GGATAGGGATAACAGGGTAATGTGCCCCAACTGGGGTAACCTTTGAGTTCTCTCAGTTGGGGGATTGTTATAACTTCGTATAGCATACATTATACGAAGTTAGCCCAGAACCTGAG   0.3%
GGATAGGGATAACAGGGTAATGTGCCCCAACTGGGGTAACCTTTGAGTTCTCTCAGTTGGGGGATTGTTATAACTTCGTATAGCA----------------------GAACCTGAG   0.2%
GGATAGGGATAACAGGGTAATGTGCCCCAACTGGGGTAACCTTTGAGTTCTC------------------------------------------------------AGAACCTGAG   0.2%

GGATCCTGGGAGGGAGAGCTTGGCAGGGGGTGGGAGGGAAGGGGGGGATGCGTGACCTGCCCGGTTCTCAGTGGCCACCCTGCGCTACCCTCTCCC           WT reference
GGAT----------------------------------------------GCGTGACCTGCCCGGTTCTCAGTGGCCACCCTGCGCTACCCTCTCCC   0.38%
GGA------------------------------------AGGGGGGGATGCGTGACCTGCCCGGTTCTCAGTGGCCACCCTGCGCTACCCTCTCCC   0.36%
G-----------------CTTGGCAGGGGGTGGGAGGGAAGGGGGGGATGCGTGACCTGCCCGGTTCTCAGTGGCCACCCTGCGCTACCCTCTCCC   0.36%   Indels
------------------CTTGGCAGGGGGTGGGAGGGAAGGGGGGGATGCGTGACCTGCCCGGTTCTCAGTGGCCACCCTGCGCTACCCTCTCCC   0.22%
```

Supplementary Fig. 8. Split circular TJ-PE mediates insertion at *AAVS1* a, Illustration of the circularization pathway to generate split circular TJ-PE. Yellow: circularization sequence. **b**, Sequencing analysis of the 100-bp insertion. All alleles observed with frequency ≥ 0.2% are shown. Green: Insertion sequence. Red: Indels.

Fig.S9

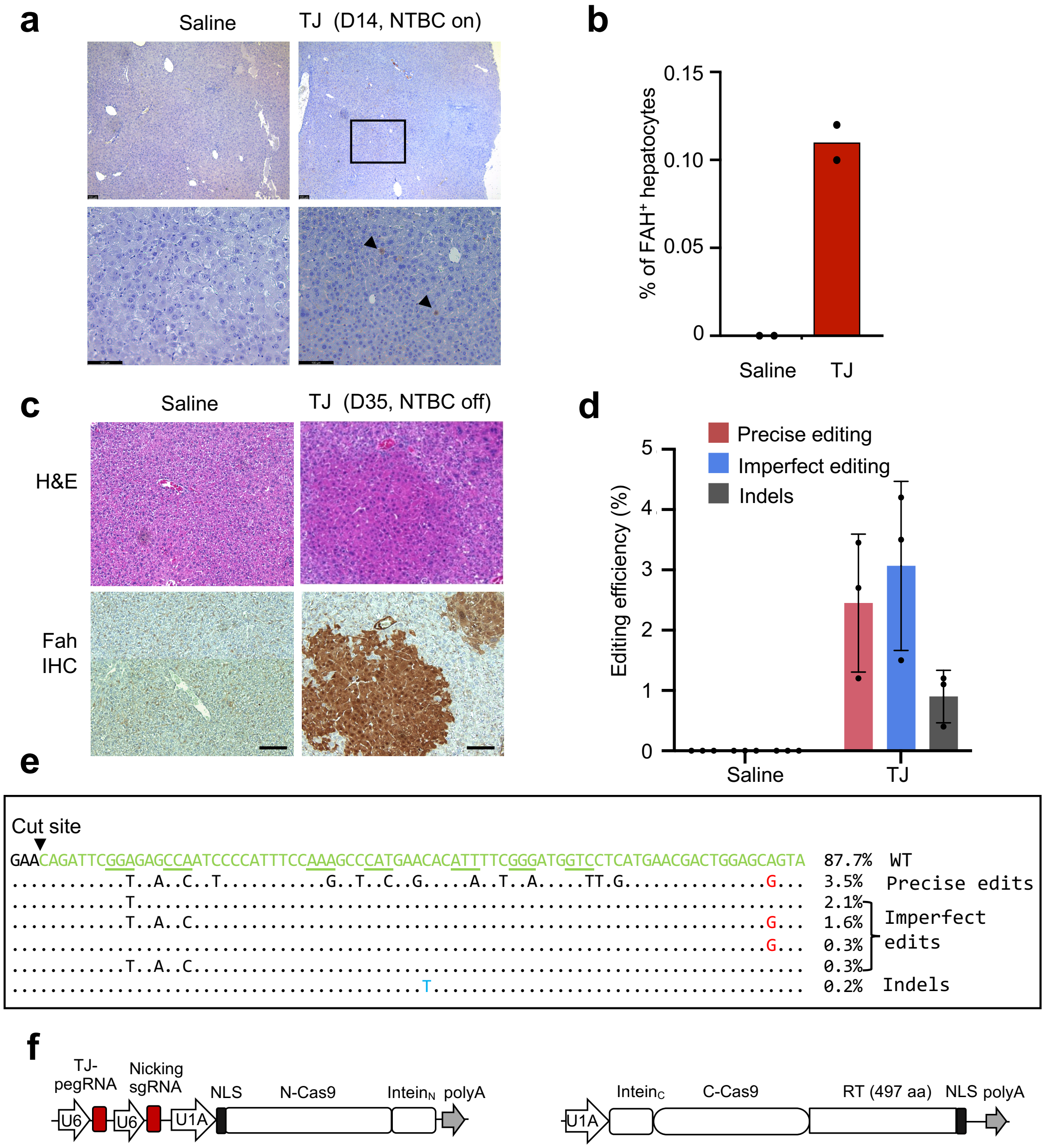

Supplementary Fig. 9. TJ-PE rewrites a correction exon in mouse liver. a, IHC staining of liver sections from *Fah*-mutant mice 14 days after TJ-PE treatment. FAH+ hepatocytes are indicated by arrows; scale bar, 100 μm. The lower panel of TJ is a high-magnification view (box with black line). Experiments were done two times, and one is shown. **b**, Quantification of FAH+ hepatocytes by IHC two weeks after hydrodynamic injection. (n=2 mice). **c**, Hematoxylin and eosin (H&E) staining and FAH Immunohistochemistry (IHC) staining of mouse liver sections 35 days after NTBC withdrawal. Experiments were done three times, and one is shown. Scale bar, 100 μm. **d**, Amplicon sequencing of exon 8 from TJ-PE-treated mouse livers two months after NTBC withdrawal. Results were obtained from three independent experiments, shown as mean ± s.d. **e**, Allele frequencies of a representative liver in **d**. All alleles ≥ 0.2% are shown. Green: Genomic sequence. Codons are underlined. Black: synonymous mutations in the correction exon. Red: correction G. Blue: indel. **f**, Schematic of the split-intein dual AAV8.

Fig.S10

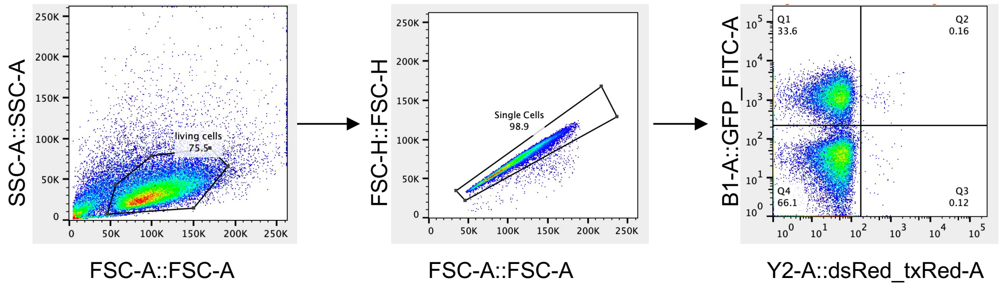

Supplementary Fig. 10. FACS gating examples for GFP-positive or Cherry-positive cells.

Supplementary Table 1. Sequences of pegRNAs and sgRNAs used in this study.
sgRNA scaffold
GTTTTAGAGCTAGAAATAGCAAGTTAAAATAAGGCTAGTCCGTTATCAACTTGAAAAAGTGGGACCGAGTCGGTCC
RC-PBS2-Insertion-PBS1-Tevopreq1

pegRNA	spacer sequence (5'-3')	3' extension
AAVS1 200 bp insertion TJ-pegRNA	GATGGAGCCAGAGAGGATCC	GCAGCTCAGGTTCTGGGATAACTTCGTATAATGTATGCTATACGA<br>AGTTATAACAATCCCCCAACTGAGAGAACTCAAAGGTTACCCCA<br>GTTGGGGCACATTACCCTGTTATCCCTACCTCGCCGGACACGCTG<br>AACTTGTGGCCGTTTACGTCGCCGTCCAGCTCGACCAGGATGGGC<br>ACCACCCCGGTGAACAGCTCCTCGCCCTTGCTCACCATTCCTCTCT<br>GGCTCTCTCTCTCTTGACGCGGTTCTATCTAGTTACGCGTTAAACC<br>AACTAGAAATTTTTTT
AAVS1 200 bp insertion pegRNA	GATGGAGCCAGAGAGGATCC	CCGGGCAGGTCACGCATATAACTTCGTATAATGTATGCTATACGA<br>AGTTATAACAATCCCCCAACTGAGAGAACTCAAAGGTTACCCCA<br>GTTGGGGCACATTACCCTGTTATCCCTACCTCGCCGGACACGCTG<br>AACTTGTGGCCGTTTACGTCGCCGTCCAGCTCGACCAGGATGGGC<br>ACCACCCCGGTGAACAGCTCCTCGCCCTTGCTCACCATTCCTCTCT<br>GGCTCTCTCTCTCTTGACGCGGTTCTATCTAGTTACGCGTTAAACC<br>AACTAGAAATTTTTTT
AAVS1 300 bp insertion TJ-pegRNA	GATGGAGCCAGAGAGGATCC	GCAGCTCAGGTTCTGGGATAACTTCGTATAATGTATGCTATACGA<br>AGTTATAACAATCCCCCAACTGAGAGAACTCAAAGGTTACCCCA<br>GTTGGGGCACATTACCCTGTTATCCCTATAGGTCAGGGTGGTCAC<br>GAGGGTGGGCCAGGGCACGGGCAGCTTGCCGGTGGTGCAGATGA<br>ACTTCAGGGTCAGCTTGCCGTAGGTGGCATCGCCCTCGCCCTCGC<br>CGGACACGCTGAACTTGTGGCCGTTTACGTCGCCGTCCAGCTCGA<br>CCAGGATGGGCACCACCCCGGTGAACAGCTCCTCGCCCTTGCTCA

		CCATTCCTCTCTGGCTCTCTCTCTTGACGCGGTTCTATCTAGTTACGCGTTAAACCAACTAGAAATTTTTTT
AAVS1 300 bp insertion pegRNA	GATGGAGCCAGAGAGGATCC	CCGGGCAGGTCACGCATATAACTTCGTATAATGTATGCTATACGAAGTTATAACAATCCCCCAACTGAGAGAACTCAAAGGTTACCCCAGTTGGGGCACATTACCCTGTTATCCCTATAGGTCAGGGTGGTCACGAGGGTGGGCCAGGGCACGGGCAGCTTGCCGGTGGTGCAGATGAACTTCAGGGTCAGCTTGCCGTAGGTGGCATCGCCCTCGCCCTCGCCGGACACGCTGAACTTGTGGCCGTTTACGTCGCCGTCCAGCTCGACCAGGATGGGCACCACCCCGGTGAACAGCTCCTCGCCCTTGCTCACCATTCCTCTCTGGCTCTCTCTCTCTTGACGCGGTTCTATCTAGTTACGCGTTAAACCAACTAGAAATTTTTTT
AAVS1 500 bp insertion TJ-pegRNA	GATGGAGCCAGAGAGGATCC	GCAGCTCAGGTTCTGGGATAACTTCGTATAATGTATGCTATACGAAGTTATAACAATCCCCCAACTGAGAGAACTCAAAGGTTACCCCAGTTGGGGCACATTACCCTGTTATCCCTACCTCCTTGAAGTCGATGCCCTTCAGCTCGATGCGGTTCACCAGGGTGTCGCCCTCGAACTTCACCTCGGCGCGGGTCTTGTAGTTGCCGTCGTCCTTGAAGAAGATGGTGCGCTCCTGGACGTAGCCTTCGGGCATGGCGGACTTGAAGAAGTCGTGCTGCTTCATGTGGTCGGGGTAGCGGCTGAAGCACTGCACGCCGTAGGTCAGGGTGGTCACGAGGGTGGGCCAGGGCACGGGCAGCTTGCCGGTGGTGCAGATGAACTTCAGGGTCAGCTTGCCGTAGGTGGCATCGCCCTCGCCCTCGCCGGACACGCTGAACTTGTGGCCGTTTACGTCGCCGTCCAGCTCGACCAGGATGGGCACCACCCCGGTGAACAGCTCCTCGCCCTTGCTCACCATTCCTCTCTGGCTCTCTCTCTTGACGCGGTTCTATCTAGTTACGCGTTAAACCAACTAGAAATTTTTTT
AAVS1 500 bp insertion pegRNA	GATGGAGCCAGAGAGGATCC	CCGGGCAGGTCACGCATATAACTTCGTATAATGTATGCTATACGAAGTTATAACAATCCCCCAACTGAGAGAACTCAAAGGTTACCCCAGTTGGGGCACATTACCCTGTTATCCCTACCTCCTTGAAGTCGATGCCCTTCAGCTCGATGCGGTTCACCAGGGTGTCGCCCTCGAACTTCACCTCGGCGCGGGTCTTGTAGTTGCCGTCGTCCTTGAAGAAGATGGTGCGCTCCTGGACGTAGCCTTCGGGCATGGCGGACTTGAAGAAGTCGTGCTGCTTCATGTGGTCGGGGTAGCGGCTGAAGCACTGCACGCCGTAGGTCAGGGTGGTCACGAGGGTGGGCCAGGGCACGGGCAGCTTGCCGGTGGTGCAGATGAACTTCAGGGTCAGCTTGCCGTAGGTGGCATCGCCCTCGCCCTCGCCGGACACGCTGAACTTGTGGCCGTTTACGTCGCCGTCCAGCTCGACCAGGATGGGCACCACCCCGGTGAACAGCTCCTCGCCCTTGCTCACCATTCCTCTCTGGCTCTCTCTCTTGACGCGGTTCTATCTAGTTACGCGTTAAACCAACTAGAAATTTTTTT
HEK3 200 bp insertion TJ-pegRNA	GGCCCAGACTGAGCACGTGA	GTCAACCAGTATCCCGGATGGTGAGCAAGGGCGAGGAGCTGTTCACCGGGGTGGTGCCCATCCTGGTCGAGCTGGACGGCGACGTAAACGGCCACAAGTTCAGCGTGTCCGGCGAGGGCGAGGGCGATGCCACCTACGGCAAGCTGACCCTGAAGTTCATCTGCACCACCGGCAAGCTGCCCGTGCCCTGGCCCACCCTCGTGACCACCCTGACCTACGTGCTCAGTCTGTAGACACACGCGGTTCTATCTAGTTACGCGTTAAACCAACTAGAATTTTTTT
HEK3 200 bp insertion control PBS2 pegRNA	GGCCCAGACTGAGCACGTGA	CCTTTCCTCTGCCATCAATGGTGAGCAAGGGCGAGGAGCTGTTCACCGGGGTGGTGCCCATCCTGGTCGAGCTGGACGGCGACGTAAACGGCCACAAGTTCAGCGTGTCCGGCGAGGGCGAGGGCGATGCCACCTACGGCAAGCTGACCCTGAAGTTCATCTGCACCACCGGCAAGCTGCCCGTGCCCTGGCCCACCCTCGTGACCACCCTGACCTACGTGCTCAGTCTGTAGACACACGCGGTTCTATCTAGTTACGCGTTAAACCAACTAGAATTTTTTT
PRNP 200 bp insertion TJ-pegRNA	GCAGTGGTGGGGGGCCTTGG	GCATGTTTTCACGATAGATGGTGAGCAAGGGCGAGGAGCTGTTCACCGGGGTGGTGCCCATCCTGGTCGAGCTGGACGGCGACGTAAACGGCCACAAGTTCAGCGTGTCCGGCGAGGGCGAGGGCGATGCCA

		CCTACGGCAAGCTGACCCTGAAGTTCATCTGCACCACCGGCAAGC TGCCCGTGCCCTGGCCCACCCTCGTGACCACCCTGACCTAAGGCC CCCCACCATCTCTCTCTTGACGCGGTTCTATCTAGTTACGCGTTAA ACCAACTAGAAATTTTTTT
PRNP 200 bp insertion pegRNA	GCAGTGGT GGGGGGCC TTGG	CCAGCATGTAGCCGCCAATGGTGAGCAAGGGCGAGGAGCTGTTC ACCGGGGTGGTGCCCATCCTGGTCGAGCTGGACGGCGACGTAAA CGGCCACAAGTTCAGCGTGTCCGGCGAGGGCGAGGGCGATGCCA CCTACGGCAAGCTGACCCTGAAGTTCATCTGCACCACCGGCAAGC TGCCCGTGCCCTGGCCCACCCTCGTGACCACCCTGACCTAAGGCC CCCCACCATCTCTCTCTTGACGCGGTTCTATCTAGTTACGCGTTAA ACCAACTAGAAATTTTTTT
IDS 200 bp insertion TJ-pegRNA	GCATTTTC GATTCCGT GACT	ACTGAGGGATGTCTGAAATGGTGAGCAAGGGCGAGGAGCTGTTC ACCGGGGTGGTGCCCATCCTGGTCGAGCTGGACGGCGACGTAAA CGGCCACAAGTTCAGCGTGTCCGGCGAGGGCGAGGGCGATGCCA CCTACGGCAAGCTGACCCTGAAGTTCATCTGCACCACCGGCAAGC TGCCCGTGCCCTGGCCCACCCTCGTGACCACCCTGACCTACACGG AATCGAAATCTCTCTCTTGACGCGGTTCTATCTAGTTACGCGTTAA ACCAACTAGAAATTTTTTT
IDS 200 bp insertion pegRNA	GCATTTTC GATTCCGT GACT	CGGATCCTCTTCCAAGTATGGTGAGCAAGGGCGAGGAGCTGTTCA CCGGGGTGGTGCCCATCCTGGTCGAGCTGGACGGCGACGTAAAC GGCCACAAGTTCAGCGTGTCCGGCGAGGGCGAGGGCGATGCCAC CTACGGCAAGCTGACCCTGAAGTTCATCTGCACCACCGGCAAGCT GCCCGTGCCCTGGCCCACCCTCGTGACCACCCTGACCTACACGGA ATCGAAATCTCTCTCTTGACGCGGTTCTATCTAGTTACGCGTTAAA CCAACTAGAAATTTTTTT
CCR5 400 bp Insertion TJ-pegRNA	CTCACTAT GCTGCCGC CCAG	GGTACCTATCGATTGTCATGGTGAGCAAGGGCGAGGAGCTGTTCA CCGGGGTGGTGCCCATCCTGGTCGAGCTGGACGGCGACGTAAAC GGCCACAAGTTCAGCGTGTCCGGCGAGGGCGAGGGCGATGCCAC CTACGGCAAGCTGACCCTGAAGTTCATCTGCACCACCGGCAAGCT GCCCGTGCCCTGGCCCACCCTCGTGACCACCCTGACCTACGGCGT GCAGTGCTTCAGCCGCTACCCCGACCACATGAAGCAGCACGACTT CTTCAAGTCCGCCATGCCCGAAGGCTACGTCCAGGAGCGCACCAT CTTCTTCAAGGACGACGGCAACTACAAGACCCGCGCCGAGGTGA AGTTCGAGGGCGACACCCTGGTGAACCGCATCGAGCTGAAGGTA GGGATAACAGGGTAATGGCGGCAGCATAGTCTCTCTCTTGACGCG GTTCTATCTAGTTACGCGTTAAACCAACTAGAAATTTTTTT
IDS 400 bp Insertion TJ-pegRNA	GCATTTTC GATTCCGT GACT	ACTGAGGGATGTCTGAAATGGTGAGCAAGGGCGAGGAGCTGTTC ACCGGGGTGGTGCCCATCCTGGTCGAGCTGGACGGCGACGTAAA CGGCCACAAGTTCAGCGTGTCCGGCGAGGGCGAGGGCGATGCCA CCTACGGCAAGCTGACCCTGAAGTTCATCTGCACCACCGGCAAGC TGCCCGTGCCCTGGCCCACCCTCGTGACCACCCTGACCTACGGCG TGCAGTGCTTCAGCCGCTACCCCGACCACATGAAGCAGCACGACT TCTTCAAGTCCGCCATGCCCGAAGGCTACGTCCAGGAGCGCACCA TCTTCTTCAAGGACGACGGCAACTACAAGACCCGCGCCGAGGTG AAGTTCGAGGGCGACACCCTGGTGAACCGCATCGAGCTGAAGGT AGGGATAACAGGGTAATCACGGAATCGAAATCTCTCTCTTGACGC GGTTCTATCTAGTTACGCGTTAAACCAACTAGAAATTTTTTT
PRNP 400 bp Insertion TJ-pegRNA	GCAGTGGT GGGGGGCC TTGG	GCATGTTTTCACGATAGATGGTGAGCAAGGGCGAGGAGCTGTTC ACCGGGGTGGTGCCCATCCTGGTCGAGCTGGACGGCGACGTAAA CGGCCACAAGTTCAGCGTGTCCGGCGAGGGCGAGGGCGATGCCA CCTACGGCAAGCTGACCCTGAAGTTCATCTGCACCACCGGCAAGC TGCCCGTGCCCTGGCCCACCCTCGTGACCACCCTGACCTACGGCG TGCAGTGCTTCAGCCGCTACCCCGACCACATGAAGCAGCACGACT TCTTCAAGTCCGCCATGCCCGAAGGCTACGTCCAGGAGCGCACCA TCTTCTTCAAGGACGACGGCAACTACAAGACCCGCGCCGAGGTG AAGTTCGAGGGCGACACCCTGGTGAACCGCATCGAGCTGAAGGT

		AGGGATAACAGGGTAATAGGCCCCCACCATCTCTCTCTTGACGC<br>GGTTCTATCTAGTTACGCGTTAAACCAACTAGAAATTTTTTT
GFP 89 bp replacement TJ-pegRNA	AGTTCAGC<br>GTGTCCGG<br>CTT	GTCAGGGTGGTCACGAGCGTAGGCCAGGGAACAGGCAGCTTACC<br>CGTTGTGCAAATGAATTTGAGTGTGAGTTTCCCATAGGTGGCATC<br>GCCCTCGCCCTCGCCGGACACGCTGATAAATATCTTGACGCGGTT<br>CTATCTAGTTACGCGTTAAACCAACTAGAAATTTTTTT
SA-Puro insertion TJ-pegRNA	GATGGAGC<br>CAGAGAG<br>GATCC	GCAGCTCAGGTTCTGGGTCAGGCACCGGGCTTGCGGGTCATGCAC<br>CAGGTGCGCGGTCCTTCGGGCACCTCGACGTCGGCGGTGACGGTG<br>AAGCCGAGCCGCTCGTAGAAGGGGAGGTTGTGGGGCGCGGAGGT<br>CTCCAGGAAGGCGGGCACCCCGGCGCGCTCGGCCGCCTCCACTCC<br>GGGGAGCACGACGGCGCTGCCCAGACCCTTGCCCTGGTGGTCGG<br>GCGAGACGCCGACGGTGGCCAGGAACCACGCGGGCTCCTTGGGC<br>CGGTGCGGCGCCAGGAGGCCTTCCATCTGTTGCTGCGCGGCCAGC<br>CGGGAACCGCTCAACTCGGCCATGCGCGGGCCGATCTCGGCGAA<br>CACCGCCCCCGCTTCGACGCTCTCCGGCGTGGTCCAGACCGCCAC<br>CGCGGCGCAGTCGTCCGCGACCCACACCTTGTCGATGTCGAGCCC<br>GACGCGCGTGAGGAAGAGTTCTTGCAGCTCGGTGACCCGCTCGAT<br>GTGGCGGTCCGGATCGACGGTGTGGCGCGTGGCGGGGTAGTCGG<br>CGAACGCGGCGGCGAGGGTGCGTACGGCCCTGGGGACGTCGTCG<br>CGGGTGGCGAGGCGCACCGTGGGCTTGTACTCGGTCATTGGGCCA<br>GGATTCTCCTCGACGTCACCGCATGTTAGCAGACTTCCTCTGCCCT<br>CTCCGCTGCCAGATCTCTCGAGGCCCTGTGGGAGGAAGAGAAGA<br>GGTCAGAAGCTTTCCTCTCTGGCTCTCTCTCTCTTGACGCGGTTCT<br>ATCTAGTTACGCGTTAAACCAACTAGAAATTTTTTT
SA-GFP insertion TJ-pegRNA	GATGGAGC<br>CAGAGAG<br>GATCC	GCAGCTCAGGTTCTGGGCTCAGAATTCCTTGTACAGCTCGTCCAT<br>GCCGAGAGTGATCCCGGCGGCGGTCACGAACTCCAGCAGGACCA<br>TGTGATCGCGCTTCTCGTTGGGGTCTTTGCTCAGGGCGGACTGGG<br>TGCTCAGGTAGTGGTTGTCGGGCAGCAGCACGGGGCCGTCGCCG<br>ATGGGGGTGTTCTGCTGGTAGTGGTCGGCGAGCTGCACGCTGCCG<br>TCCTCGATGTTGTGGCGGATCTTGAAGTTCACCTTGATGCCGTTCT<br>TCTGCTTGTCGGCCATGATATAGACGTTGTGGCTGTTGTAGTTGTA<br>CTCCAGCTTGTGCCCCAGGATGTTGCCGTCCTCCTTGAAGTCGAT<br>GCCCTTCAGCTCGATGCGGTTCACCAGGGTGTCGCCCTCGAACTT<br>CACCTCGGCGCGGGTCTTGTAGTTGCCGTCGTCCTTGAAGAAGAT<br>GGTGCGCTCCTGGACGTAGCCTTCGGGCATGGCGGACTTGAAGA<br>AGTCGTGCTGCTTCATGTGGTCGGGGTAGCGGCTGAAGCACTGCA<br>CGCCGTAGGTCAGGGTGGTCACGAGGGTGGGCCAGGGCACGGGC<br>AGCTTGCCGGTGGTGCAGATGAACTTCAGGGTCAGCTTGCCGTAG<br>GTGGCATCGCCCTCGCCCTCGCCGGACACGCTGAACTTGTGGCCG<br>TTTACGTCGCCGTCCAGCTCGACCAGGATGGGCACCACCCCGGTG<br>AACAGCTCCTCGCCCTTGCTCACTGGGCCAGGATTCTCCTCGACG<br>TCACCGCATGTTAGCAGACTTCCTCTGCCCTCTCCGCTGCCAGATC<br>TCTCGAGGCCCTGTGGGAGGAAGAGAAGAGGTCAGAAGCTTTCC<br>TCTCTGGCTCTCTCTCTCTTGACGCGGTTCTATCTAGTTACGCGTT<br>AAACCAACTAGAAATTTTTTT
AAVS1 split circular TJ-pegRNA		AGACCCTCGACCGTCGATTGTCCACTGGTCAACAATAGATGACTT<br>ACAACTAATCGGAAGGTGCAGAGACTCGACGGGAGCTACCCTAA<br>CGTCAAGACGAGGGTAAAGAGAGAGTCCAATTCTCAAAGCCAAT<br>AGGCAGTAGCGAAAGCTGCAAGAGAATGAAAATCCGTTGACCTT<br>AAACGGTCGTGTGGGTTCAAGTCCCTCCACCCCCACGCCGGAAAC<br>GCAATAGCCGAAAAACAAAAAAGCACATGAGGATCACCCATGTG<br>CGCAGCTCAGGTTCTGGGATAACTTCGTATAATGTATGCTATACG<br>AAGTTATAACAATCCCCCAACTGAGAGAACTCAAAGGTTACCCC<br>AGTTGGGGCACATTACCCTGTTATCCCTATCCTCTCTGGCTCCATC<br>GTAAGCAAACCTTAGAGGTTCTGGCAAGCAAAAAAACAAAACGG<br>CTATTATGCGTTACCGGCGAGACGCTACGGACTTAAATAATTGAG

		CCTTAAAGAAGAAATTCTTTAAGTGGATGCTCTCAAACTCAGGGAAACCTAAATCTAGTTATAGACAAGGCAATCCTGAGCCAAGCCGAAGTAGTAATTAGTAAGACCAGTGGACAATCGACGGATAACAGCATATCTAG
Fah TJ-pegRNA-1	GTAGGCCCTGGGAACAGATT	TAAGAACAGAACATCAGAGGAAGCTGGGCCACCAGGCATTACCGCTCCAGTCGTTCATCAAAACCATTCCAAATATGTGCTCGTGAGCCTTGGAAATGGGAATGGGTTCACCGAATCTGTTCCCAGGGCTATATATCTAGACGCGGTTCTATCTAGTTACGCGTTAAACCAACTAGAATTTTTTT
Fah TJ-pegRNA-2	GTAGGCCCTGGGAACAGATT	TCAGAGGAAGCTGGGCCACCACATTACCGCTCCAATCATTCATCAAAACCATTCCAAATATATGCTCGTGAGCCTTGGAGATCGGAATGGGTTCACCAAACCTGTTCCCAGGGCTATATATCTAGACGCGGTTCTATCTAGTTACGCGTTAAACCAACTAGAATTTTTTT

Nicking sgRNA	spacer sequence (5'-3')
AAVS1 nicking sgRNA	GCAGCTCAGGTTCTGGGAGA
HEK3 nicking sgRNA	GTCAACCAGTATCCCGGTGC
HEK3 control nicking sgRNA	GCACATACTAGCCCCTGTCT
PRNP nicking sgRNA	GCATGTTTTCACGATAGTAA
IDS nicking sgRNA	ACTGAGGGATGTCTGAAGGC
TLR reporter nicking sgRNA	TAGGTCAGGGTGGTCACGA
Fah nicking sgRNA-1	CCCTAAGAACAGAACATCAG
Fah nicking sgRNA-2	CAGAGGAAGCTGGGCCACC

Supplementary Table 2. Sequences of primers used for genomic DNA amplification.

	**F (5'-3')**	**R (5'-3')**
AAVS1	CTTGCCAGAACCTCTAAGGT	CCAGGATCAGTGAAACGCAC
HEK3	TCTGCTGCAAGTAAGCATGCATTTG	GCCCCTTCCAGGGACCTC
PRNP	AGTAAGCCAAAAACCAACATG	CTGTACTCATCCATGGGCCT
IDS	ACGTTGAGCTGTGCAGAGAA	GTGCGTATGGAATAGCCCAT

Supplementary Table 3. Sequences of primers used for ddPCR.

**Gene**	**F (5'-3')**	**R (5'-3')**	**Probe**
AAVS1	CTTGCCAGAACCTCTAAGGT	GCTGAACTTGTGGCCGTTT	Ins CACCACCCCGGTGAACAGCTC
HEK3	GCATGCATTTGTAGGCTTGATG	CAGCCAAACTTGTCAACCAG	WT CCTGGCCTGGGTCAATCCTTGG<br>Ins CACGGGCAGCTTGCCGGTGG<br>Ins CACCACCCCGGTGAACAGCTC
PRNP	F GTCAGTGGAACAAGCCGAGT<br>F TAGGTCAGGGTGGTCACGAG	ACTTGGTTGGGGTAAC<br>GGTG	WT TGAAGCACATGGCTGGTGCTGC<br>Ins CACGGGCAGCTTGCCGGTGG<br>Ins CACCACCCCGGTGAACAGCTC
IDS	F GCTGAACTTGTGGCCGTTT<br>F TAGGTCAGGGTGGTCACGAG	GTGCGTATGGAATAGCCCAT	Ins CACCACCCCGGTGAACAGCTC<br>Ins CACGGGCAGCTTGCCGGTGG

CCR5	TAGGTCAGGGTGGT CACGAG	TTCCTGGGAGAGACG CAAAC	Ins CACGGGCAGCTTGCCGGTGG

Supplementary Table 4. Sequences of primers used for high throughput sequencing.

Gene	F (5'-3')	R (5'-3')
AAVS1	AGACGTGTGCTCTTCCGATCTCTTGCC AGAACCTCTAAGGT	CTACACGACGCTCTTCCGATCTCCAGGAT CAGTGAAACGCAC
Fah	CTACACGACGCTCTTCCGATCTACCAA CTTTCTCCATGGCAG	AGACGTGTGCTCTTCCGATCTTGTCCCAT ACCCAACTCCTG
