# Supplementary material for: Template-jumping prime editing enables large insertion and exon rewriting in vivo
Source: Nat Commun. 2023 Jun 8;14:3369. doi: 10.1038/s41467-023-39137-6 (PMC10250319; doi:10.1038/s41467-023-39137-6)
Supplement: Supplementary file 2 — Reporting Summary [file 41467_2023_39137_MOESM2_ESM.pdf]

nature portfolio

Corresponding author(s): Wen Xue, Erik J. Sontheimer

Last updated by author(s): May 23, 2023

Reporting Summary

Nature Portfolio wishes to improve the reproducibility of the work that we publish. This form provides structure for consistency and transparency in reporting. For further information on Nature Portfolio policies, see our Editorial Policies and the Editorial Policy Checklist.

Statistics

For all statistical analyses, confirm that the following items are present in the figure legend, table legend, main text, or Methods section.

n/a	Confirmed	
☐	☒	The exact sample size (*n*) for each experimental group/condition, given as a discrete number and unit of measurement
☐	☒	A statement on whether measurements were taken from distinct samples or whether the same sample was measured repeatedly
☒	☐	The statistical test(s) used AND whether they are one- or two-sided<br>*Only common tests should be described solely by name; describe more complex techniques in the Methods section.*
☒	☐	A description of all covariates tested
☒	☐	A description of any assumptions or corrections, such as tests of normality and adjustment for multiple comparisons
☐	☒	A full description of the statistical parameters including central tendency (e.g. means) or other basic estimates (e.g. regression coefficient) AND variation (e.g. standard deviation) or associated estimates of uncertainty (e.g. confidence intervals)
☒	☐	For null hypothesis testing, the test statistic (e.g. *F*, *t*, *r*) with confidence intervals, effect sizes, degrees of freedom and *P* value noted<br>*Give P values as exact values whenever suitable.*
☒	☐	For Bayesian analysis, information on the choice of priors and Markov chain Monte Carlo settings
☒	☐	For hierarchical and complex designs, identification of the appropriate level for tests and full reporting of outcomes
☒	☐	Estimates of effect sizes (e.g. Cohen's *d*, Pearson's *r*), indicating how they were calculated

Our web collection on statistics for biologists contains articles on many of the points above.

Software and code

Policy information about availability of computer code

Data collection	Illumina Miseq, Bio-Rad ddPCR
Data analysis	CRISPResso V2.0.32 was used to analyze deep data for quantifying editing efficiency. Frequency, mean, and standard deviations were calculated using GraphPad Prism 8. Flow cytometry data were analyzed by FlowJo 10.0 software.

For manuscripts utilizing custom algorithms or software that are central to the research but not yet described in published literature, software must be made available to editors and reviewers. We strongly encourage code deposition in a community repository (e.g. GitHub). See the Nature Portfolio guidelines for submitting code & software for further information.

Data

Policy information about availability of data

All manuscripts must include a data availability statement. This statement should provide the following information, where applicable:

- Accession codes, unique identifiers, or web links for publicly available datasets
- A description of any restrictions on data availability
- For clinical datasets or third party data, please ensure that the statement adheres to our policy

A reporting summary for this article is available as a Supplementary Information file. The raw sequencing data have been deposited in the NCBI BioProject database under accession code PRJNA973981.

Human research participants

Policy information about studies involving human research participants and Sex and Gender in Research.

Reporting on sex and gender	No human research participants
Population characteristics	No human research participants
Recruitment	No human research participants
Ethics oversight	No human research participants

Note that full information on the approval of the study protocol must also be provided in the manuscript.

Field-specific reporting

Please select the one below that is the best fit for your research. If you are not sure, read the appropriate sections before making your selection.

☒ Life sciences ☐ Behavioural & social sciences ☐ Ecological, evolutionary & environmental sciences

For a reference copy of the document with all sections, see nature.com/documents/nr-reporting-summary-flat.pdf

Life sciences study design

All studies must disclose on these points even when the disclosure is negative.

Sample size	1x105 cells were used for editing in culture system. All cell samples were evaluated in at least biological triplicates (n = 3) to ensure the reproductability. Our previous editing studies have shown that this sample size and replications are sufficient to ensure reproducibility (Liu et al, Nature Biotechnology; Jiang et al, Nature Biotechnology; Song et al, Nature Biomedical Engineering,2019 and Ibraheim et al, Nature Communications, 2021).
Data exclusions	No data was excluded.
Replication	All the culture-related experiments were done in biological triplicate in culture cells, n=3 ,on different days (every three days). All attempts at replication were successful, and standard deviations were in the expected ranges.
Randomization	After seeding cell into 24-well plate, we randomly decided which well is for experiment group or control group. Group allocation for animal study was performed randomly.
Blinding	It is not applied to molecular and cell experiments. Groups were labeled. All mouse work are blind.

Reporting for specific materials, systems and methods

We require information from authors about some types of materials, experimental systems and methods used in many studies. Here, indicate whether each material, system or method listed is relevant to your study. If you are not sure if a list item applies to your research, read the appropriate section before selecting a response.

Materials & experimental systems

n/a	Involved in the study	
☐	☒	Antibodies
☐	☒	Eukaryotic cell lines
☒	☐	Palaeontology and archaeology
☐	☒	Animals and other organisms
☒	☐	Clinical data
☒	☐	Dual use research of concern

Methods

n/a	Involved in the study	
☒	☐	ChIP-seq
☐	☒	Flow cytometry
☒	☐	MRI-based neuroimaging

Antibodies

Antibodies used	Fumarylacetoacetate hydrolase antibody (ab83770, Abcam Inc), IHC 1:400
Validation	The specificity of the anti-Fah antibody has previously been confirmed(Yin et al, Nature Biotech,2016).

Eukaryotic cell lines

Policy information about cell lines and Sex and Gender in Research

Cell line source(s)	HEK293T cells (ATCC) and HEK293T-TLR cells, A549 (ATCC), U-2 OS (ATCC)
Authentication	HEK293T, A549 and U-2 OS cells were acquired from ATCC validated by supplier (ATCC) by STR analysis. And HEK293T-TLR cells were validated using specific primers to amplify the inserted cassette.
Mycoplasma contamination	All cell lines tested negative for mycoplasma contamination
Commonly misidentified lines (See ICLAC register)	The cell lines used in this article are not in the list of misidentified lines.

Animals and other research organisms

Policy information about studies involving animals; ARRIVE guidelines recommended for reporting animal research, and Sex and Gender in Research

Laboratory animals	4-8 weeks transgenic Fah mutant mice were used in this study.
Wild animals	No wild animals were used in the study.
Reporting on sex	Female and male mice were used in this study.
Field-collected samples	No field collected samples were used in the study.
Ethics oversight	All animal study protocols were approved by the UMass IACUC

Note that full information on the approval of the study protocol must also be provided in the manuscript.

Flow Cytometry

Plots

Confirm that:

- [x] The axis labels state the marker and fluorochrome used (e.g. CD4-FITC).
- [x] The axis scales are clearly visible. Include numbers along axes only for bottom left plot of group (a 'group' is an analysis of identical markers).
- [x] All plots are contour plots with outliers or pseudocolor plots.
- [x] A numerical value for number of cells or percentage (with statistics) is provided.

Methodology

Sample preparation	Cell lines were detached and performed Flow cytometry analysis immediately.
Instrument	MACSQuant
Software	FlowJo
Cell population abundance	No purfication
Gating strategy	The cells were first gated based on FSC/FSCH to select for live single cells. Unedited TLR cells were employed as negative control.

- [x] Tick this box to confirm that a figure exemplifying the gating strategy is provided in the Supplementary Information.
